# Personality and peer groups in adolescence: Reciprocal associations and shared genetic and environmental influences

**DOI:** 10.1111/jopy.12741

**Published:** 2022-07-27

**Authors:** D. Angus Clark, C. Emily Durbin, Mary M. Heitzeg, William G. Iacono, Matt McGue, Brian M. Hicks

**Affiliations:** ^1^ Department of Psychiatry University of Michigan Ann Arbor Michigan USA; ^2^ Department of Psychology Michigan State University East Lansing Michigan USA; ^3^ Department of Psychology University of Minnesota Minneapolis Minnesota USA

**Keywords:** adolescence, behavior genetics, heritability, peer behavior, personality, shared environmental influences

## Abstract

**Objective:**

Peer groups represent a critical developmental context in adolescence, and there are many well‐documented associations between personality and peer behavior at this age. However, the precise nature and direction of these associations are difficult to determine as youth both select into, and are influenced by, their peers.

**Method:**

We thus examined the phenotypic, genetic, and environmental links between antisocial and prosocial peer characteristics and several personality traits from middle childhood to late adolescence (ages 11, 14, and 17 years) in a longitudinal twin sample (*N* = 3762) using teacher ratings of personality and self‐reports of peer characteristics.

**Results:**

Less adaptive trait profiles (i.e., high negative emotionality, low conscientiousness, and low agreeableness) were associated with more antisocial and fewer prosocial peer characteristics across time. Associations between personality traits related to emotionality (negative emotionality and extraversion) and peer behavior were largely attributable to shared genetic influences, while associations between personality traits related to behavioral control (conscientiousness and agreeableness) and peer behavior were due to overlapping genetic and shared environmental influences.

**Conclusions:**

Overall, results suggest a set of environmental presses that push youth toward both behavioral undercontrol and antisocial peer affiliations, making the identification of such influences and their relative importance a critical avenue of future work.

## 1 INTRODUCTION

Individual differences in orientation toward others (e.g., approach vs. withdrawal), perceptions about others, and how individuals understand themselves in relation to others, play a central role in most models of personality. In adolescence, peers become the dominant “others” as adolescents become especially attuned to their connections and status among peers, both in dyadic relationships and broader social networks (Brown & Klute, 2006; Hartup & Stevens, 1997), heightening the potential for peer processes to shape behavior and identity during a key period of personality development. While connections between personality traits and peer relationship metrics during adolescence are numerous, the causal direction of these associations is difficult to parse as people both select into and is influenced by their peer networks (i.e., there are bidirectional effects between individuals and peer groups) (Coplan & Bullock, 2012; Prinstein & Giletta, 2016; Wrzus & Neyer, 2016). Here, we used a longitudinal twin sample to help further our understanding of these connections by examining the phenotypic, genetic, and environmental associations between personality traits and peer characteristics during adolescence, a critical developmental window in which individuals are working to solidify an identity in the context of a rapidly evolving social world. We explored how the behavioral ecology of youth's peer groups transacts with the development of their traits over time. Specifically, rather than focusing on relationship qualities youth experience (such as satisfaction, closeness, or degree of conflict) that could be conceptualized as reflecting or emerging from personality traits of youth themselves (such as individual differences in interpersonal warmth or antagonism), we explored how youth personality traits shaped and are influenced by the behavioral characteristics of their friends, specifically the degree to which they perceived their friends as engaging in broad patterns of antisocial and prosocial behavior.

### 1.1 Co‐development of personality traits and peer characteristics in adolescence

During adolescence, peers and romantic partners become the dominant social relationships as opposed to parents and family (Brown & Klute, 2006; Hartup & Stevens, 1997). Relative to children, adolescents spend more time with their peers than parents, disclose more with friends, and place a higher value on their peers' evaluations and perceive greater risks of being excluded from peer groups and friendships or losing status within peer groups (Brown, 2011; Brown & Larson, 2009; Furman & Buhrmester, 1992) that corresponds to a peak in behavioral conformity with group norms (Laursen & Veenstra, 2021). As youth become more integrated with their peers, these peer groups also grow larger and more heterogeneous (e.g., more cross‐sex friendships) than those of childhood (Brown, 1999). Peer relationships thus become more challenging and uncertain to navigate while feeling more psychologically consequential. This salience gives peer relationships the potential to serve as key drivers of developmental change in other systems, including identity, values, and behavior. Thus, peer relationships are an important source of developmental pressure in adolescence that could prompt subsequent change in personality traits. Compared to emerging adulthood, during which social norms facilitate normative trajectories of personality maturation (Bleidorn, 2015), the behavioral ecology of adolescence permits and incentivizes a broader range of behavior, including a normative increase in deviant behaviors (e.g., Farrington, 1986; Monahan et al., 2009). This context provides an important window characterized by increasing variance in adolescence, both in dispositions (e.g., Mõttus et al., 2019) and peer group behavior, to examine for mechanisms of person‐environment transactions.

However, the qualities of peer relationships are likely also impacted by the personality traits youth bring to and express in the peer context. For example, youth report large increases in both their own, and their friends', antisocial behaviors during adolescence (Farrington, 2004). This is consistent with observed changes in self‐reported traits, with early to mid‐adolescents describing themselves as more impulsive, less compliant with rules and obedient to authority, less agreeable (Denissen et al., 2013; Soto et al., 2011) and for girls, more neurotic (Durbin et al., 2016; Soto, 2015). The co‐developmental process of affiliation with peers that engage in more antisocial behavior, and seeing oneself as having less well‐adjusted personality traits, raises questions about how the social ecology of adolescent friendships shape the development of personality traits and related outcomes.

### 1.2 Mechanisms linking personality and peer relationships in adolescence

#### 1.2.1 Selection effects

One obvious possibility is that friendship networks are formed partially on the basis of homophily and attraction effects (McPherson et al., 2001; Neal et al., 2014), namely, that people socialize preferentially and form relationships based on concordant behaviors and personality structures. That is, young people choose or seek out friend relationships and networks on the basis of fit between their traits and the characteristics of their peers, and are recruited into those groups and relationships by others on the basis of their traits. The net result of selection and recruitment effects is that individual differences in traits will be correlated with the behavioral characteristics of friends and the friendship network. For example, groups of adolescents with high levels of antisocial behavior are expected to form because youth with the personality structures associated with antisocial behavior (i.e., impulsivity and aggression) will select into relationships with others who have similar traits and behaviors (Burk et al., 2007; Gallupe et al., 2020). Consequently, the aggregation of people with a personality structure that leans toward antisocial behavior will result in a network of friends with increasingly similar levels of antisocial behaviors over time.

If genetic influences contribute to these traits and behaviors, this will result in a “genetic control of the construction of experiences” (Kandler & Zapko‐Willmes, 2017) whereby these heritable individual differences will be magnified because these differences shape and constrain the environments in which youth develop. Stability of these traits will also be impacted by selection effects, as youth will largely have friends who are more similar than not to them (i.e., homophily), providing a context that will tend to affirm the characteristics that are shared among members of the group via both norms and explicit reinforcement, thus discouraging mean level changes on those traits within persons but potentially heightening differences across people interacting with different peer groups.

### 1.3 Influence effects

As a result of their salience in adolescence, peers likely have a unique ability to shape behavior and personality through a variety of mechanisms (Cruz et al., 2012; Prinstein & Giletta, 2016; Rubin et al., 2013). First, behavioral norms may be created and reinforced through exposure to behaviors among close others and the imitation of those normative behaviors. Second, friends can create environments that facilitate certain behaviors; for example, youth who have initiated substance use and have access to substances will make it easier for their peers to initiate use, and will convey more positive attitudes about initiation (Dishion & Owen, 2002; Robin & Johnson, 1996). Third, peers may explicitly punish and reinforce specific behaviors (Laninga‐Wijnen & Veenstra, 2021). To the extent that peer groups provide important opportunities for self‐disclosure, social support, identity exploration, and romantic relationships, the interpersonal and affective climate of peer relationships may play an especially pivotal role in shaping youth behavioral characteristics. Because friendship relationships are self‐selected (compared to classmates, for example), they would be expected to exert more powerful effects on development (Zimmermann & Reitz, 2018). Friendships that come together partially because of shared experiences may provide climates of even greater feelings of support and mutual understanding (e.g., Lodder et al., 2016), enhancing the ability of friends to subsequently shape one another's behaviors and traits. Pure influence effects are present when someone behaves or thinks in a way that they might not have in the absence of exposure and interaction with particular others (Laursen & Veenstra, 2021). In the case of personality development, the presence of selection effects requires longitudinal data to disentangle selection from interaction effects, as it permits assessment of traits and their development as unfolding before, during, and after selection into different kinds of peer groups.

### 1.4 Environmental reinforcement of individual differences and corresponsive specificity

Finally, the corresponsive principle of development states that the traits that lead a person to select into an environment are the very ones that will be most shaped by exposure to that environment (Roberts et al., 2003). For example, impulsive young people select into friendships with similar peers, and processes within their co‐created peer environment reinforce individual dispositions toward impulsivity, thus widening individual differences in traits across youth over time (Cairns et al., 1988; Dishion et al., 1995, 1996). This suggests that rather than general effects of peer group qualities on all traits, observed impacts will be greater on those traits that predict selection as well. Moreover, one would expect enhancement of genetic effects on traits as environments operate to reinforce and exacerbate genetically mediated individual differences, with this canalization effect accumulating over time (Briley et al., 2019). For example, relationships with more antisocial peers would be expected to provide more opportunities for youth disposed toward similar behavior to engage in and have such behaviors rewarded. By contrast, when youth do change on personality traits, it may create divergence across friends that weakens their connection to one another, potentially ending those relationships (Brown & Larson, 2009).

### 1.5 Genetic and environmental influences

The potential mechanisms linking personality traits and peer characteristics entail different patterns of genetic and environmental influence. In behavioral genetic models, variation in a given trait is typically decomposed into additive genetic (*a* ^2^), common or shared environmental (*c* ^2^), and nonshared environmental (*e* ^2^) variance components (Prescott, 2004). The additive genetic variance component captures the extent to which individual differences are due to genetic differences between people, while the shared environmental variance component captures environmental forces that increase familial similarity, and the nonshared environmental variance component captures environmental forces (including nonsystematic measurement error) that decrease familial similarity.

Both personality traits *and* peer group characteristics are heritable, that is, a nontrivial amount of the individual variability in these traits is due to genetic influences (Clark et al., 2020; Cruz et al., 2012; Johnson et al., 2008; Prinstein & Giletta, 2016). In the case of peer group characteristics, this suggests a general degree of selection as youths' own genetic makeup cannot directly affect the behavior of their peers. This is consistent with the idea of an active gene–environment correlation (rGE; Scarr & McCartney, 1983) in which a person's inherited propensities make them more likely to seek out certain environments. However, both friend characteristics and personality traits (especially earlier in life) are also influenced by environmental factors (Burt, 2014; Clark et al., 2017, 2020; Cruz et al., 2012). This leaves open the possibility that associations between personality traits and peer group characteristics can be explained by shared genetic influences (consistent with selection effects), environmental influences (consistent with peer influence effects, or broader environmental presses toward both certain traits and peer affiliations), or transactions between genetic and environmental factors. That is, the critical question is the extent to which the associations between personality traits and peer group characteristics are explained by genetic versus environmental factors. Longitudinal, developmental designs are especially important for understanding the processes that give rise to observed estimates of the relative importance of genetic and environmental causes, as they allow for examination of interplay between genetic and environmental factors over time (Briley et al., 2018), such as those that typify transactions between adolescent dispositions and the makeup of peer groups in which adolescents accomplish many of the key developmental tasks of this period.

### 1.6 Current study

We used a longitudinal, genetically informative design to explore associations between personality and peer characteristics over time, and the source of those associations. This allowed us to rule out heritable influences masquerading as environmental effects due to the heritability of environments via selection processes, and to identify shared environmental influences that are important to adolescent development. That is, to the extent associations between personality traits and peer characteristics are explained by genetic influences, it suggests a degree of selection such that youth choose to affiliate with certain peer groups as a function of their own inherited dispositions (e.g., toward impulsive behavior). Alternatively, to the extent the association between personality traits and peer behaviors is explained by environmental influences, it suggests that peer behavior may exert an external press on personality (or that broader environmental forces push youth toward both certain dispositions and peer groups).

To examine these effects, we used the random intercept cross‐lagged panel model (RI‐CLPM; Hamaker et al., 2015), a modeling approach that allowed us to more effectively isolate between person‐stable trait variance and between‐person influences from within‐person effects. It was then possible to identify genetic and environmental influences on the stable between‐person variance over time. Our ability to make inferences about personality and peer characteristics was further bolstered by a multi‐informant approach. Specifically, we assessed personality via teacher ratings of traits, which we have previously shown have unique predictive validity for important aspects of youth functioning (Clark et al., 2017), and ratings by participants of their peers' characteristics. This independence of the peer and personality measures produces estimates of the effects of interest that are decontaminated by bias from shared informants.

## 2 METHOD

### 2.1 Participants

The sample consisted of 3762 (52% female) twins (1881 pairs) from the Minnesota Twin Family Study (MTFS), an ongoing longitudinal developmental study of psychosocial health and adjustment (Iacono et al., 1999; Keyes et al., 2009). Twin pairs met the following criteria: were the same sex, living within driving distance to the University of Minnesota laboratories and with at least one biological parent at the time of recruitment, and were free of any cognitive or physical disability that would interfere with study assessments. Twins were recruited the year they turned either 11 years old (*n* = 2510; the younger cohort; born between 1977 and 1994) or 17 years old (*n* = 1252; the older cohort; born between 1972 and 1979), and were invited to return for follow‐up assessments every 3–5 years. Families were representative of the geographical area from which they were drawn in terms of socioeconomic status, history of mental health treatment, and urban versus rural residence (Iacono et al., 1999). Consistent with the demographics of Minnesota for the target birth years, 96% of participants reported White non‐Hispanic race and ethnicity.

All twin pairs were assessed at age 17 (*M*_age_ = 17.85 years; *SD* = .64 years), but only the younger cohort was assessed at ages 11 (*M*_age_ = 11.78 years; *SD* = .43 years) and 14 (*M*_age_ = 14.90 years; *SD* = .31 years). Thus, attrition is not the source of smaller sample sizes at ages 11 and 14. Attrition was low in the younger cohort (retention rates were 91.4% and 86.3% at ages 14 and 17, respectively). In terms of zygosity, there were 395 male and 394 female monozygotic (MZ) twin pairs, and 220 male and 246 female dizygotic (DZ) twin pairs at age 11; 364 male and 370 female MZ twin pairs, and 203 male and 230 female DZ twin pairs at age 14; 529 male and 582 female MZ twin pairs, and 299 male and 331 female DZ twin pairs at age 17. Zygosity was originally assessed by parents' reports on a standard zygosity questionnaire and staff evaluations of physical similarity of eyes, hair, face, ears, and fingerprint ridge counts (Iacono et al., 1999). Zygosity was later confirmed by genome‐wide genotyping (McGue et al., 2013).

### 2.2 Personality

Personality traits were assessed using 28 items from a teacher rating form completed by up to three teachers nominated by the twin participants. Items consisted of short descriptors (Table 1) that were designed as markers of the primary scales of the Multidimensional Personality Questionnaire (Tellegen & Waller, 2008) and other trait constructs. For each item, teachers were instructed to rank the participant in reference to their classmates as falling in either the lowest 5%, lower 30%, middle 30%, higher 30%, or highest 5% of students in class. The mean rating across teachers was used whenever more than one teacher rating was available (~75% of participants with teacher rating data had at least two teacher informants). Teacher ratings were collected at each assessment and were available for 91.2%, 84.2%, and 78.2% of participants at ages 11, 14, and 17, respectively. It was Minnesota state policy to place members of twin pairs in separate classrooms whenever possible, which minimizes any bias due to twin contrast or comparison.

**TABLE 1:** TRF items, affiliated traits, and standardized factor loadings across time

Personality item	Age 11	Age 14	Age 17
Cheerful, interested, optimistic^e^	.79	.78	.78
Persuasive, dominant, socially visible^e^	.71	.76	.76
Ambitious, hard driving, persevering^c^	.88	.88	.86
Gregarious, people‐oriented, warm^e^	.87	.87	.87
Tense, sensitive, moody^n^	.75	.76	.77
Feels treated poorly, exploited, unlucky^n^	.87	.88	.86
Tough, unforgiving, aggressive^n^	.67	.61	.60
Careful. level‐headed, planful^c^	.82	.85	.82
Thrill seeking, adventurous, risk‐taking^e^	.41	.37	.29
Absorbed, fascinated, image‐prone	–	–	–
Values a good reputation, endorses strictness, respects authority^a^	.87	.86	.84
Athletic	–	–	–
Popular, well liked^e^	.79	.79	.79
Intelligent^c^	.81	.81	.81
Charming with the opposite sex	–	–	–
Entertaining, funny^e^	.78	.76	.75
Mature, acts and appears older^c^	.78	.76	.78
Experience with alcohol or drugs	–	–	–
Truthful, trustworthy^a^	.92	.90	.89
Confident^c^	.82	.79	.75
Law abiding^a^	.86	.87	.87
Artistic	–	–	–
Nuturant, helpful, concerned about others^a^	.76	.83	.70
Miserable, unhappy, distressed^n^	.77	.77	.80
Superficial, shallow	–	–	–
A Leader	–	–	–
Nervous, fearful, timid	–	–	–
Manipulative^a^	.67	.68	.64

*Note*: Superscripts denote which trait (if any) items were associated with in the main analyses (e = extraversion, n = negative emotionality, c = conscientiousness, a = agreeableness). Factor structure based on preliminary exploratory factor analyses; factor loadings come from single factor confirmatory factor analysis in which the mean and variance of the latent factor were fixed to 0 and 1, respectively. Standardized factor loadings presented.

We analyzed the psychometric structure of the items from the teacher rating form to streamline analyses and increase generalizability to the existing literature, which tends to emphasize broad trait dimensions (e.g., the Big Five). We submitted the 28 items to exploratory factor analyses (EFA) separately for data from ages 11, 14, and 17. Oblique geomin rotation was used, and a parallel analysis with 1000 random draws was run with each EFA. For each age, a four‐factor solution was best supported based on examinations of the scree plot, the results of parallel analyses, and the psychological interpretability of the factors.

The factors roughly corresponded to the extraversion/positive affect, conscientiousness, neuroticism/negative emotionality, and agreeableness/prosociality constructs in Big Five trait models of personality. After identifying these target constructs we refined scale measures by excluding items that did not load well on any factor (i.e., *λ* < .40; e.g., “artistic”), had high cross‐loadings across factors that were inconsistent across ages (e.g., “nervous, fearful, timid” loaded on both negative emotionality and extraversion, but to different extents across ages), or did not seem relevant to personality (e.g., “athletic”).

Table 1 lists the final item set organized by scale. The four trait scales had high internal consistency reliability (Cronbach's *α*), and high average factor loadings (*λ*) from one‐factor confirmatory factor analytic models fit at ages 11, 14, and 17: Extraversion (*α* = .86, mean *λ* = .86), Negative Emotionality (*α* = .76, mean *λ* = .84), Conscientiousness (*α* = .91, mean *λ* = .82), Agreeableness (*α* = .89, mean *λ* = .80). Scale scores were calculated by taking the mean of the items; scores could range between 1 and 5.

### 2.3 Friend characteristics

Twins rated the proportion of their friends (1 = *none of my friends are like that*, 2 = *just a few of my friends are like that*, 3 = *most of my friends are like that*, 4 = *all of my friends are like that*) that exhibited various types of antisocial (breaks the rules; gets in trouble with the police; get in fights; gets in trouble at school; steals; drinks alcohol; smokes cigarettes; uses drugs; 9 items, *α* = .85) and prosocial (gets good grades in school; are very smart; popular with other kids; good at sports; does their homework and studies a lot; liked by teachers; 9 items, *α* = .85) behaviors and attributes (Walden et al., 2004) at each assessment. Scale scores were calculated by taking the sum of the items; scores could range between 9 and 36. Twin reports of friend characteristics were not part of the baseline assessment for the male twins of the older cohort and male twins from the earlier birth years for the younger cohort (1977–1982). Consequently, the number of reports on friend characteristics are lower at ages 11 and 17 than would be anticipated given the baseline sample sizes and rates of attrition.

### 2.4 Data analytic strategy

**FIGURE 1:**
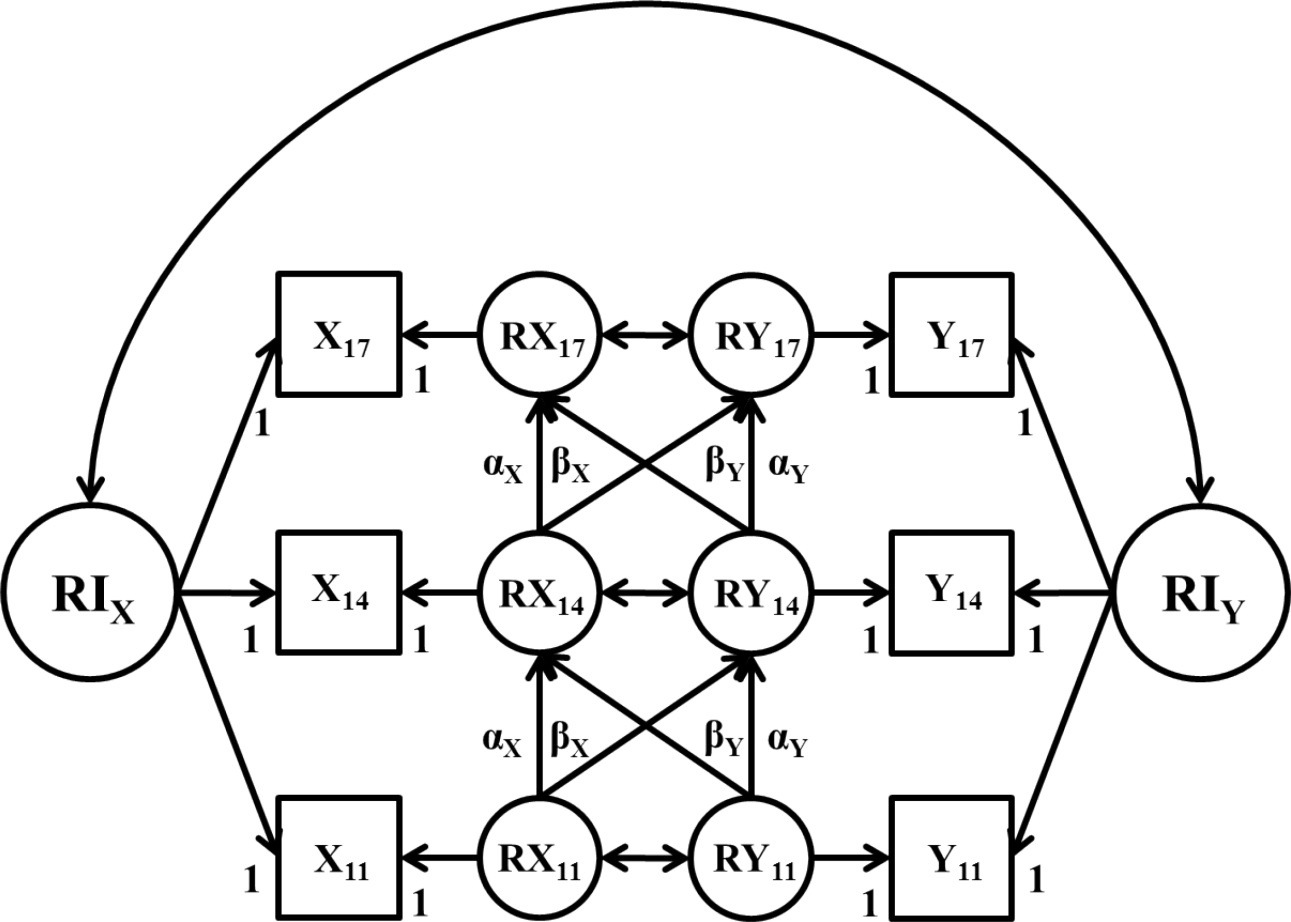
Random intercept cross‐lagged panel model (RI‐CLPM). RI = random intercept; R_11_ − R_17_ = residuals factors at age 11 through age 17; *α* = autoregressive paths; *β* = cross lag paths. Variances and mean structure omitted from figure for clarity of presentation.

Zero‐order correlations between teacher reports of youth personality traits and youth informant reports of perceived antisocial and prosocial characteristics of their friends were initially examined both within and between time points. RI‐CLPMs were then used to examine longitudinal associations between personality traits and friend characteristics (see Figure 1). Each pairing of a personality trait and either antisocial or prosocial friend characteristics was analyzed in turn. In these models, assessments of personality (denoted as *X*_11_, *X*_14_, *X*_17_ in Figure 1) and peer behavior (denoted as *Y*_11_, *Y*_14_, *Y*_17_ in Figure 1) at each time point were each specified to load on construct‐specific random intercept factors (RI in Figure 1). The random intercept factors capture participants' overall level of the latent trait over time as estimated by the observed data across waves (similar to an individual's mean‐level across time relative to the sample norm). Factor loadings were fixed to 1, and indicator intercepts were allowed to vary. The mean of the random intercept was fixed to 0 and the variance was freely estimated. Occasion‐specific residual factors (*RX*_11_ through *RY*_17_ in Figure 1) were then specified. Factor loadings on the residual factors were fixed to 1, the means of the residual factors were fixed to 0, and the residual factor variances were freely estimated. Autoregressive paths were added from one residual factor to the next subsequent residual factor (*α*_*x*_ and α_y_ in Figure 1). Cross‐lagged paths were also specified between the residual factors over time (*β*_*x*_ and *β*_*y*_ in Figure 1). Within‐occasion covariances between the personality and friend behavior residual factors were also specified, as was a covariance between the personality and friend behavior random intercepts. In this model, the cross‐lagged paths (*β*_*x*_ and *β*_*y*_ in Figure 1) capture associations between personality and friend behavior at the within‐person level (i.e., the extent to which occasion‐specific deviations from an individual's trend of scores across time points are correlated over time) while the correlation between the two random intercept factors captures associations between personality and friend behavior at the between‐person level (i.e., the extent to which stable individual differences across time points in a personality trait and friend characteristics are correlated).

We then estimated the genetic and environmental contributions to the associations between personality and friend characteristics. Univariate ACE models were first fit for the personality and peer characteristics assessments at each occasion, as well as for the random intercept and residual structure of univariate random intercept models (Prescott, 2004). In ACE models, intraclass correlations between twins for MZ and DZ twins are used to parse the phenotypic variance into additive genetic (*a* ^2^), common or shared environmental (*c* ^2^), and nonshared environmental (*e* ^2^; i.e., environmental influences that contribute to differences among relatives) variance components.

**FIGURE 2:**
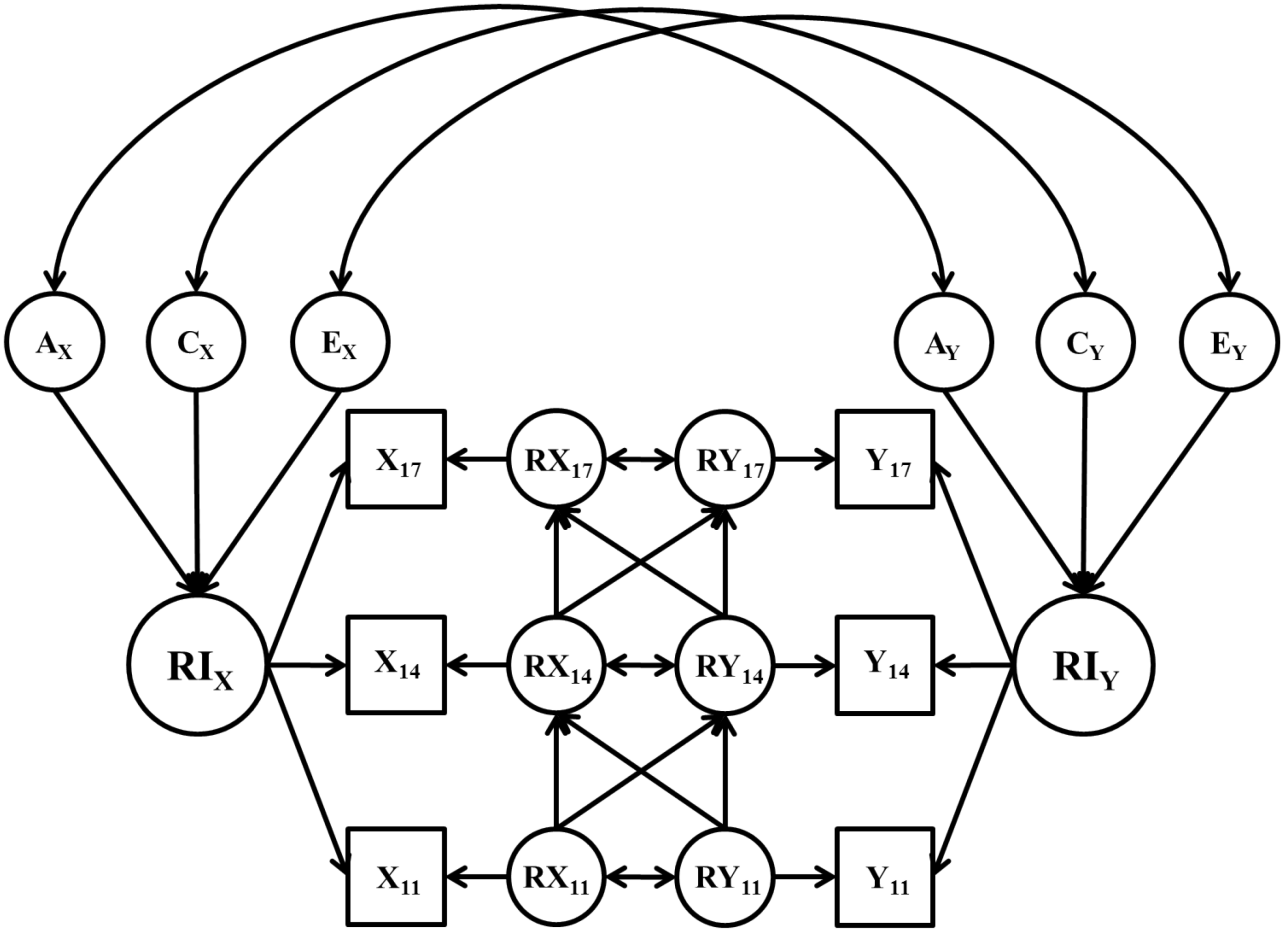
Conceptual diagram of the correlated ace random intercept cross‐lagged panel model (RI‐CLPM). RI = random intercept; *R*_11_ − *R*_17_ = residuals factors at age 11 through age 17; A = additive genetic variance components; C = shared environmental variance components; E = nonshared environmental variance components. Twin 2 model not pictured. Two equivalent models were specified, one for twin 1 and one for twin 2, and covariances were added between the twin 1 and 2 A and C variance components. The covariance between the A components was fixed to either 1.0 (MZ twins) or 0.5 (DZ twins), and the covariance between the C components was fixed to 1.0 (MZ and DZ twins).

Following the univariate ACE models biometric versions of the RI‐CLPM were fit (see conceptual path diagram in Figure 2). To estimate the ACE correlations depicted in Figure 2, we first had to fit a model that implied a (causal) direction to the association between a personality trait and aspect of friend behavior (antisocial or prosocial characteristics), even though that direction is arbitrary in this context. We then use the results of these models to calculate the ACE correlations. For these directional models, variance in the friend behavior random intercept was specified as a function of its own ACE components only. The personality random intercept, however, was specified as a function of the genetic and environmental influences that overlap with friend behavior (i.e., friend behavior random intercept ACE components), and genetic and environmental influences on personality that are independent of friend behavior (i.e., residual ACE components on personality random intercept). Because we cannot infer causality in this design, the path estimates from the directional model were converted to bi‐directional ACE correlations that does not imply a causal direction (Figure 2) to quantify the overlap between genetic and environmental influences across constructs (traits and friend characteristics), and the extent to which that overlap explained the observed phenotypic correlation (i.e., how well does the genetic covariance between a trait and a peer characteristic account for the observed covariance between the measures of these constructs).

To facilitate the estimation of the bivariate models, the ACE path estimates for the peer characteristics random intercept were fixed to the estimates derived from the univariate random intercept ACE models, with ACE components explaining less than 10% of the total variance in the univariate models being fixed to 0 in this part of the model (e.g., if there were no additive genetic influences on the antisocial friend behaviors random intercept then no overlap in additive genetic influence was estimated between antisocial friend behaviors and personality). Genetic and environmental overlap was not examined in the residual structure as models including these paths encountered serious convergence difficulties. Although a limitation, ACE components are inherently between‐person constructs, making them better suited conceptually for examining between‐person associations.

All models were fit in Mplus version 8.4 (Muthen & Muthen, 2020) using full information maximum likelihood (FIML) estimation. The phenotypic and behavioral genetic models were run using a nonparametric percentile bootstrap procedure with 1000 draws (clustered by family for the phenotypic analyses), which provides reliable confidence intervals for assessing parameter estimate precision under a variety of complex data conditions (Falk, 2018). A parameter estimate was considered statistically significant if the bootstrapped 95% confidence interval did not include 0. The Mplus Automation Package (Hallquist & Wiley, 2018) in R (R Development Core Team, 2016) was also used to facilitate the analyses.

## 3 RESULTS

### 3.1 Preliminary analyses

Descriptive information for all of the study variables is reported in Table 2. Mean differences across time in the personality traits were small. There was moderate rank‐order stability across time in teacher reports of youth personality traits (*r*s from .30 to .61; mean *r* = .49), with negative emotionality being the least stable across time. From ages 11 to 17 there was a large mean‐level increase in youth reports of their friends' antisocial behaviors (*d* = .75), and a small decrease in youth reports of their friends' prosocial behaviors (*d* = −.31). Friends' antisocial and prosocial behaviors also exhibited moderate rank‐order stability across time that was similar in magnitude to the stability for teacher reports of youth traits (*r*s from .36 to .55; mean *r* = .43).

**TABLE 2:** Descriptive information for personality traits and peer behavior across time

	Personality				Peer behavior	
	Extraversion	Negative emotionality	Conscientiousness	Agreeableness	Antisocial peers	Prosocial peers
Age 11						
*M*	3.21	1.74	3.39	3.94	10.59	25.18
*SD*	.70	.71	.80	.77	1.98	3.48
*N*	2292	2291	2291	2291	1684	1681
Age 14						
*M*	3.20	1.70	3.53	3.96	13.51	23.40
*SD*	.70	.67	.77	.73	4.13	3.32
*N*	1932	1928	1933	1929	2259	2252
*r*11	.52	.37	.61	.52	.32	.28
Age 17						
*M*	3.35	1.76	3.64	3.98	15.29	23.09
*SD*	.66	.68	.72	.70	4.26	3.07
*N*	2672	2662	2672	2670	2673	2666
*r*11	.47	.30	.56	.46	.29	.25
*r*14	.54	.37	.59	.53	.56	.48

Abbreviations: M, mean; N, number of ratings; *r*11, correlation with corresponding variable at age 11; *r*14, correlation with corresponding variable at age 14; SD, standard deviation.

Zero‐order correlations between teacher‐reported youth personality traits and youth reports of their friends' behaviors are reported in Table 3. Friends' antisocial behaviors were consistently associated with youth negative emotionality (mean *r* within and between time points = .26), lower conscientiousness (mean *r* within and between time points = −.25), and lower agreeableness (mean *r* within and between time points = −.39), but were unrelated to extraversion (mean *r* within and between time points = −.02). Friends' prosocial behaviors were consistently associated with youth's own extraversion (mean *r* within and between time points = .24), conscientiousness (mean *r* within and between time points = .25), and agreeableness (mean *r* within and between time points = .24), and lower negative emotionality (mean *r* within and between time points = −.19). Overall, this indicates that greater personality adjustment in youth is broadly associated with youth reports that their friends engage in more prosocial behaviors and fewer antisocial behaviors in adolescence.

**TABLE 3:** Phenotypic correlations between personality traits and peer behavior

	Peer behavior at Age 11		Peer behavior at age 14		Peer behavior at age 17	
	Antisocial peers	Prosocial peers	Antisocial peers	Prosocial Peers	Antisocial peers	Prosocial peers
Personality at age 11						
Extraversion	.00	**.27**	−.01	**.20**	.02	**.21**
Negative emotionality	**.38**	**−.23**	.16	**−.20**	.19	−.17
Conscientiousness	**−.21**	**.24**	−.19	**.22**	**−.20**	**.23**
Agreeableness	**−.45**	**.23**	**−.30**	**.21**	**−.33**	.19
Personality at age 14						
Extraversion	−.06	**.21**	−.02	**.27**	−.02	**.23**
Negative emotionality	**.25**	−.16	**.35**	**−.23**	**.23**	−.17
Conscientiousness	**−.26**	**.22**	**−.32**	**.30**	**−.27**	**.23**
Agreeableness	**−.35**	.17	**−.48**	**.31**	**−.38**	**.21**
Personality at age 17						
Extraversion	−.08	.19	−.01	**.24**	−.02	**.35**
Negative emotionality	**.20**	−.14	**.25**	−.19	**.32**	**−.23**
Conscientiousness	**−.20**	**.20**	**−.27**	**.24**	**−.36**	**.36**
Agreeableness	**−.34**	**.20**	**−.34**	**.27**	**−.53**	**.35**

*Note*: Correlation coefficients greater than or equal to |.20| presented in bold.

### 3.2 Phenotypic associations over time

Fit information for the RI‐CLPMs is reported in Table 4. Most models fit the data well on the basis of conventional standards for judging absolute model fit (e.g., RMSEA < .08, CFI > .90, TLI > .90, SRMR < .08; West et al., 2012). The exception was the model for friends' antisocial behavior and youth agreeableness, which did not appear to fit well on the basis of three of the five indices considered. An examination of the modification indices and residuals suggested that—consistent with the large zero‐order correlations between these measures reported in Table 3—the cross‐lagged paths, within‐time covariances, and random intercept covariance together still underestimated the extent of the overlap between friends' behaviors and agreeableness. Given the limited degrees of freedom (*df* = 1 for all models), desire to keep the models consistent, and lack of conceptual impact, the original model was retained, however. That is, the lack of fit for this model primarily indicates that friends' antisocial behaviors and agreeableness overlap considerably, such that even after accounting for the dynamics modeled here there remains additional covariation between the assessments of the two constructs at each time point that could be modeled if more degrees of freedom were available (though restraint would of course still be necessary to avoid over‐specifying models for the purpose of chasing better fit).

**TABLE 4:** Model fit information for random intercept cross‐lagged panel models

	*χ* ^2^	*df*	*p*	RMSEA	CFI	TLI	SRMR
Antisocial peers							
Extraversion	3.67	1	.06	.027	.999	.981	.008
Negative emotionality	14.49	1	<.01	.06	.992	.881	.015
Conscientiousness	5.726	1	.017	.036	.998	.975	.010
Agreeableness	43.52	1	<.01	.107	.985	.771	.027
Prosocial peers							
Extraversion	5.13	1	.02	.033	.998	.969	.012
Negative emotionality	2.21	1	.14	.018	.999	.986	.008
Conscientiousness	8.86	1	<.01	.046	.997	.952	.018
Agreeableness	1.21	1	.27	.007	1.00	.998	.007

Abbreviations: CFI, comparative fit index (values above .90 indicate adequate fit); *df*, model degrees of freedom; *p*, p value for chi square test of exact fit; RMSEA, root mean square error of approximation (values below .08 indicate adequate fit); SRMR, standardized root mean square residual (values less than .08 indicate adequate fit); TLI, Tucker Lewis Index (values above .90 indicate adequate fit); *χ* ^2^, chi square model test statistic.

Standardized autoregressive and cross‐lagged path estimates from the RI‐CLPMs are reported in Table 5. Generally speaking, scores on the residual factors were more stable over time from ages 11 to 14 for personality (mean |*β*| = .17 vs. .10), and ages 14 to 17 for friend behaviors (mean |*β*| = .40 vs. .08). That is, youth scoring higher than implied by the random intercept and occasion‐specific intercepts at one occasion were also more likely to score higher at the subsequent occasion. For personality this relative stability was more pronounced earlier in adolescence, whereas for friend behaviors this relative stability was more pronounced later in adolescence. These differences in relative stability across individual dispositions and the characteristics of youths' peer groups could be the result of the differential developmental context of the early versus middle to late adolescence stages captured across these two intervals. Youths' peer group ecology may become less fluid with age. Individual dyads and groups may stabilize as adolescents commit to particular relationships or their identity formation encourages stabilization of peer groups. Reputational factors may reduce mobility across groups that vary in their behavioral norms. As a result, over time, it may be more difficult to move across very different peer groups, producing greater stability in these measures of peer group characteristics.

**TABLE 5:** Standardized path coefficients from random intercept cross‐lagged panel models

	Antisocial peers				Prosocial peers			
	Extraversion	Negative emotionality	Conscientiousness	Agreeableness	Extraversion	Negative emotionality	Conscientiousness	Agreeableness
*Autoregressive Paths* *(α in Figure* *1* *)*								
Personality → Personality								
Age 11 → Age14	**.16** **[.06, .25]**	**.13** **[.02, .23]**	**.23** **[.12, .32]**	**.25** **[.13, .35]**	**.17** **[.07, .26]**	**.12** **[.01, .22]**	**.20** **[.09, .30]**	**.13** **[.01, .25]**
Age 14 → Age17	**.14** **[.02, .25]**	.07 [−.05, .20]	.02 [−.14, .16]	.13 [−.04, .28]	**.14** **[.02, .25]**	.10 [−.01, .22]	.07 [−.07, .20]	.09 [−.06, .22]
Peers → Peers								
Age 11 → Age14	.06 [−.24, .24]	.05 [−.26, .23]	.07 [−.24, .24]	.05 [−.22, .22]	.08 [−.01, .18]	.09 [−.01, .18]	.11 [.00, .21]	.10 [.00, .19]
Age 14 → Age17	**.50** **[.44, .55]**	**.49** **[.43, .55]**	**.47** **[.40, .53]**	**.44** **[.38, .51]**	**.31** **[.21, .39]**	**.32** **[.22, .40]**	**.31** **[.22, .40]**	**.32** **[.22, .41]**
*Cross‐Lagged Paths* *(β in Figure* *1* *)*								
Personality → Peers								
Age 11 → Age14	.00 [−.09, .08]	**.13** **[.04, .20]**	**−.18** **[−.26, −.05]**	**−.26** **[−.34, −.15]**	**.10** **[.01, .19]**	**−.10** **[−.18, −.03]**	**.13** **[.02, .22]**	**.13** **[.03, .21]**
Age 14 → Age17	.00 [−.06, .06]	−.01 [−.06, .06]	**−.08** **[−.15, −.02]**	**−.10** **[−.17, −.03]**	.04 [−.04, .11]	.02 [−.06, .09]	−.02 [−.10, .07]	−.02 [−.10, .06]
Peers → Personality								
Age 11 → Age14	−.04 [−.18, .09]	.08 [−.10, .21]	−.02 [−.15, .16]	.03 [−.13, .25]	.00 [−.08, .09]	.03 [−.07, .12]	−.04 [−.13, .06]	−.06 [−.17, .03]
Age 14 → Age17	.02 [−.06, .10]	**.17** **[.07, .26]**	**−.23** **[−.36, −.10]**	**−.22** **[−.36, −.09]**	.10 [.00, .19]	−.08 [−.16, .01]	.09 [−.04, .21]	**.15** **[.04, .27]**
*Random Intercept* *Correlations*								
Personality RI ←→ Peers RI	−.02 [−.16, .12]	**.29** **[.12, .48]**	**−.31** **[−.42, −.21]**	**−.49** **[−.65, −.35]**	**.37** **[.21, .53]**	**−.40** **[−.64, −.22]**	**.38** **[.24, .56]**	**.38** **[.23, .57]**

*Note*: 95% confidence intervals (derived via clustered, nonparametric percentile bootstrap with 1000 draws) presented in brackets below estimates; bolded estimates are those for which the 95% confidence interval does not include 0.

In terms of cross‐lagged paths across time, higher youth neuroticism, lower conscientiousness, and lower agreeableness at age 11 predicted higher levels of friends' perceived antisocial behaviors at age 14 (mean |*β*| = .19). Low conscientiousness and low agreeableness at age 14 also predicted greater levels of friends' antisocial behaviors at age 17, but the effects were notably reduced (mean |*β*| = .09). Friends' antisocial behaviors at age 11 were unrelated to personality at age 14. However, greater levels of friends' antisocial behaviors at age 14 predicted higher negative emotionality, lower conscientiousness, and lower agreeableness at age 17 (mean |*β*| = .21).

Extraversion, low negative emotionality, conscientiousness, and agreeableness at age 11 all predicted higher levels of friends' prosocial behaviors at age 14 (mean |*β*| = .12). The analogous paths from age 14 to 17 were trivial in magnitude. Friends' perceived prosocial behaviors at ages 11 and 14 were largely unrelated to subsequent personality scores, and the only statistically significant path from mid to late adolescence was that friends' prosocial behaviors at age 14 were associated with greater agreeableness at age 17 (*β* = .15). Overall, the consistent temporal asymmetry in the cross‐lagged paths imply that pre‐adolescent personality traits help select youth into friend groups with certain characteristics in mid‐adolescence, and these peer group characteristics (especially antisociality) in turn help to shape personality in late adolescence.

The correlations between the random intercept factors are reported in Table 5 with most associations being medium to large in magnitude. The random intercept of friends' perceived antisocial behaviors was associated with negative emotionality, low conscientiousness and low agreeableness (mean |*r*| = .36), and unrelated to extraversion. The random intercept of friends' perceived prosocial behaviors was associated with extraversion, low negative emotionality, conscientiousness, and agreeableness (mean |*r*| = .38). Overall, these results indicate that stable individual differences in personality traits reflecting positively evaluated behavioral dispositions (conscientiousness and agreeableness) are moderately to strongly associated with youth reports of their friends' antisocial (−) and prosocial (+) behaviors, while emotionality traits (neuroticism but especially extraversion) are more strongly related to prosocial friend characteristics. This pattern is notable given that the traits were assessed by teachers, for whom conscientiousness and agreeableness may be particularly salient dispositions within the educational context.

### 3.3 Genetic and environmental influences

The results from the univariate ACE models are presented in Table 6. Variability in the observed personality trait scores was primarily accounted for by the additive genetic and nonshared environmental variance components (average *a* ^2^ = .56, average *c* ^2^ = .11, average *e* ^2^ = .32). Nontrivial shared environmental influences were also detected for conscientiousness and agreeableness at ages 11 and 14, and negative emotionality at age 14. This finding contrasts with conclusions that environmental influences on personality traits are unique rather than shared in kind (e.g., Polderman et al., 2015). It is possible that our method (teacher reports) may be detecting an effect that does not emerge in self‐report data, but has been demonstrated for observational measures of traits (Borkenau et al., 2001). Variation in the random intercept factors was primarily explained by either the genetic variance component exclusively (extraversion and neuroticism), or the genetic and shared environmental variance components (conscientiousness and agreeableness) (mean *a* ^2^ = .80, mean *c* ^2^ = .18, mean *e* ^2^ = .03). Nonshared environmental influences on the random intercepts were negligible, consistent with the idea that by modeling the shared variance across time, unsystematic, time‐point specific measurement error is eliminated. Consequently, this suggests there are few nonshared environmental influences that are unrelated to unsystematic measurement error on the random intercepts. With only two exceptions—negative emotionality and agreeableness at age 14—variance in the residual factors were primarily attributed to genetic and nonshared environmental influences (mean *a* ^2^ = .37, mean *c* ^2^ = .07, mean *e* ^2^ = .56).

**TABLE 6:** Univariate ACE estimates for raw personality traits and peer behaviors scores across ages, random intercepts, and residual structures

	Random intercept			Age 11			Age 14			Age 17		
	*a* ^2^	*c* ^2^	*e* ^2^	*a* ^2^	*c* ^2^	*e* ^2^	*a* ^2^	*c* ^2^	*e* ^2^	*a* ^2^	*c* ^2^	*e* ^2^
Personality—Raw scores												
Extraversion				.70*	.00	.30*	.72*	.00	.28*	.66*	.00	.34*
Negative emotionality				.63*	.01	.36*	.23*	.34*	.43*	.46*	.01	.53*
Conscientiousness				.70*	.10	.20*	.40*	.13	.27*	.67*	.02	.31*
Agreeableness				.53*	.24*	.23*	.35*	.37*	.28*	.63*	.08	.29*
Peer behavior—Raw scores												
Antisocial peers				.20	.51*	.29*	.16	.53*	.32*	.52*	.20*	.28*
Prosocial peers				.34*	.25*	.42*	.28*	.35*	.38*	.50*	.17*	.33*
Personality traits—Random intercept and residual structure												
Extraversion	.98*	.00	.02	.46*	.00	.54*	.45*	.00	.55*	.33*	.00	.67*
Negative emotionality	.89*	.08	.03	.51*	.00	.49*	.00	.39*	.61*	.24	.03	.73*
Conscientiousness	.76*	.21*	.03	.60*	.00	.40*	.41*	.04	.54*	.32*	.00	.68*
Agreeableness	.56*	.42*	.02	.54*	.05	.41*	.14	.33*	.53*	.45*	.00	.55*
Peer behavior—Random intercept and residual structure												
Antisocial peers	.66*	.34*	.00	.00	.00	1.00*	.16*	.44*	.40*	.58*	.00	.42*
Prosocial peers	.67*	.33*	.00	.12	.10	.78*	.22	.15	.63*	.17	.03	.81*

Abbreviations: *a* ^2^, additive genetic variance; *c* ^2^, shared environmental variance; *e* ^2^, nonshared environmental variance; Observed, ACE components for individual personality and peer behavior variables at each time point; Random intercept and residual structure, ACE components for random intercept, and time‐point specific deviations (i.e., residual factors); *, 95% confidence interval (derived via nonparametric percentile bootstrap with 1000 draws) does not include 0.

The observed scores for friends' characteristics exhibited small to medium additive genetic influences at ages 11 and 14 which increased at age 17 (mean *a* ^2^ = .33). The reverse pattern was observed for shared environmental influences, which were moderate at ages 11 and 14 and small at age 17 (mean *c* ^2^ = .34). Nonshared environmental influences were consistently moderate across time (mean *e* ^2^ = .34). Variance in the random intercept factors for friends' antisocial and prosocial behaviors were attributable to both additive genetic and shared environmental influences, with no nonshared environmental influences (mean *a* ^2^ = .67, mean *c* ^2^ = .34, mean *e* ^2^ = .00). In contrast, the occasion‐specific residual factors exhibited large nonshared environmental influences at each age, with small additive genetic and moderate shared environmental influences at ages 11 and 14, and medium to large additive genetic influences at age 17 (mean *a* ^2^ = .20, mean *c* ^2^ = .12, mean *e* ^2^ = .67).

The ACE correlations from the bivariate behavioral genetic RI‐CLPMs are reported in Table 7. There was considerable genetic overlap between the random intercept factors for youth reports of their friends' antisocial behaviors and negative emotionality (*r*_*a*_ = .74)—which explained all of the observed correlation—while there was a moderate to large degree of genetic and shared environmental overlap between the random intercepts for friends' antisocial behaviors and conscientiousness (*r*_*a*_ = .22, *r*_*c*_ = 1.00) and agreeableness (*r*_*a*_ = .64, *r*_*c*_ = 1.00). There was also considerable genetic overlap between the random intercept factors for friends' prosocial behaviors and extraversion (*r*_*a*_ = .59) and negative emotionality (*r*_*a*_ = .61)—which again explained all of the observed associations—and a moderate to large degree of both genetic and shared environmental overlap between the random intercept factors for friends' prosocial behaviors and conscientiousness (*r*_*a*_ = .24, *r*_*c*_ = 1.00) and agreeableness (*r*_*a*_ = .38, *r*_*c*_ = 1.00). Taken together, these results indicate that extraversion and negative emotionality are largely related to friend characteristics through shared genetic pathways, while environmental pathways are also critical for understanding the associations between friend characteristics, conscientiousness, and agreeableness. That is, there are similar environmental presses that contribute to both higher versus lower conscientiousness and agreeableness, and the tendency to affiliate with antisocial versus prosocial peers. Of note, overlapping shared environmental influences cannot be attributable to shared reporter variance, as traits were reported by teachers and peer characteristics were reported by youth.

**TABLE 7:** Phenotypic and etiologic correlations between personality and peer behavior random intercept factors

	Antisocial peers				Prosocial peers			
	*r*	*r*_*a*_	*r*_*c*_	*r*_*e*_	*r*	*r*_*a*_	*r*_*c*_	*r*_*e*_
Extraversion	−.02	.07 (100%)	.00 (00%)	.00 (00%)	.37	.59 (100%)	.00 (00%)	.00 (00%)
Negative emotionality	.29	.74 (100%)	.00 (00%)	.00 (00%)	−.40	.61 (100%)	.00 (00%)	.00 (00%)
Conscientiousness	−.31	.22 (33%)	1.00 (67%)	.00 (00%)	.38	.24 (37%)	1.00 (63%)	.00 (00%)
Agreeableness	−.49	.64 (50%)	1.00 (50%)	.00 (00%)	.38	.23 (28%)	1.00 (72%)	.00 (00%)

*Note*: The percentage of the observed correlation explained by genetic and environmental overlap in parentheses under genetic and environmental correlations.

Abbreviations: *r*, observed correlation between personality and peer behavior random intercept factors from phenotypic RI‐CLPMs (see Table 5); *r*_a_, additive genetic correlation; *r*_c_, shared environmental correlation; *r*_e_, nonshared environmental correlation.

## 4 DISCUSSION

Personality shapes and is shaped by the social environment, making it difficult to tease apart precisely how one influences the other. To help clarify our understanding of these processes in adolescence, we conducted a longitudinal, genetically informed analysis of the co‐development of personality traits and the antisocial and prosocial behaviors of friends during this developmental period. Overall, results highlighted how personality traits (as reported by teachers) and friend group characteristics (as reported by youth) are reciprocally related over time, and furthermore, that stable associations between personality traits and peer group characteristics across adolescence can be explained by a mixture of genetic and environmental influences. However, there were substantial differences in the direction and magnitude of these effects across the traits and peer characteristics considered.

### 4.1 Personality traits and friend behaviors across adolescence

First, we found that the associations between stable individual differences in youth personality traits and their perceptions of their friends' behaviors (i.e., the random intercepts) were strong and consistent with the known correlates of these traits. Specifically, affiliation with antisocial peers was associated with high negative emotionality, low conscientiousness, and low agreeableness. Affiliating with prosocial peers was associated with the opposite personality profile (conscientiousness, agreeableness, low negative emotionality), as well as with high extraversion. These findings suggest a general congruence between one's personality structure and aspects of one's social environment in terms of adaptive versus maladaptive qualities. That is, both antisocial peer affiliations and its personality profile are associated with more negative outcomes such as antisocial behavior, substance use problems, depression, academic difficulties, and poor parent–child relationship quality. In contrast, prosocial peer affiliations and its personality profile are associated with greater engagement in academic and extracurricular activities, more friends, better quality relationships with friends and family, and less depression and substance use problems.

Regarding the occasion specific, within person paths, we found that personality in pre‐adolescence (age 11) had a small effect on selecting into prosocial peer environments and a small to medium effect on selecting into antisocial peer environments in middle adolescence. The direction of the effect seemed to reverse over time, however, such that from middle to late adolescence the influences from reports of peer characteristics (primarily friends' antisocial behaviors) to youths' own personality traits were more pronounced while the influences of personality to friend characteristics declined. That is, affiliating with antisocial peers in middle adolescence was associated with subsequent changes toward a more maladaptive personality structure from middle to late adolescence. This suggests that experiences captured by characteristics of the peer group are working to shape the subsequent development of traits, but that trait‐based selection into peer groups with differing characteristics weakens over time, perhaps because much of the relevant sorting on these personality characteristics has taken effect. To the extent that selection continues, it may be driven by homophily factors more distal to personality. The pattern of influence was similar, though smaller, for affiliating with prosocial peers in middle adolescence, which modestly predicted a more adaptive personality structure in late adolescence. This is consistent with the environmental individualization hypothesis (Kandler et al., 2019), which asserts that over time, key environments can amplify and accentuate what were originally small differences across persons to produce larger individual differences in the characteristics impacted by those environments.

These results suggest that even after accounting for the stable aspects of personality and the behavioral ecology of friendship networks, traits and friend characteristics at one time can predict the development of the other (i.e., traits or friend characteristics) at a subsequent time point. The relative magnitude of these bidirectional influences may change depending on the broader developmental context, consistent with earlier trait‐influenced selection processes yielding to a more potent impact of the peer context on traits later in adolescence. Our findings are consisted with that of Li et al. (2011), who found that peer support became more important for predicting youth academic engagement in later compared to earlier adolescence. As some have predicted that the power of peers to shape youth should decline as they become more settled in their identity and personal preferences (Laursen & Veenstra, 2021), it will be important to test this hypothesis with data extending into later adolescence and emerging adulthood.

Overall, extraversion appeared relatively impervious to the behavioral and attitudinal climate of one's friend group, aside from a modest selection effect into having more prosocial peers. This could be attributable to high levels of extraverted behavior attracting friends who are more prosocial, academically focused, and well adjusted. Such a climate is consistent with extraversion traits and would therefore be less likely to prompt change compared to a peer environment in which there was a mismatch between youth dispositions and their peers' behaviors. The failure of individual differences in extraversion to be shaped by characteristics of the friend group may also be due to its greater absolute stability, or it may reflect its looser ties to explicit behavioral norms (compared to conscientiousness, for example). Youth who seek acceptance into peer groups but are less extraverted may focus on deepening other characteristics of theirs that will accomplish a similar goal of acceptance by the friend or group, such as being valued for their agreeableness.

Negative emotionality had modest selection effects into antisocial friend groups—and away from prosocial friend groups—and was modestly impacted by involvement with more antisocial peers. It is possible that some traits, such as high negative emotionality (or low conscientiousness), may constrain recruitment into or acceptance by particular peer groups (i.e., those with more antisocial and less prosocial peers). By virtue of this “default selection,” some youth with less adaptive trait profiles might be excluded from more desired relationships and wind up with peers who were not necessarily their preferred friends (Laninga‐Wijnen & Veenstra, 2021). As a result, the combination of their pre‐existing trait profile and the norms, interactions, and opportunities within that group further push personality development in a particular direction, such as persistent high neuroticism, and away from other potential trajectories. There were stronger bidirectional associations between conscientiousness, agreeableness, and antisocial and prosocial friend characteristics, suggesting a broader ecology of norms, behaviors, and climate within the socializing environment that facilitates corresponsive processes between these adaptive individual dispositions and the norms, reinforcement, and social cohesion processes within these prosocial peer groups.

### 4.2 Genetic and environmental influences on personality and peer affiliations

Individual differences in the stable components of teacher‐reported personality traits and youth reports of characteristics of their peer affiliations (i.e., the random intercepts) were generally explained by large genetic and moderate shared environmental influences. The random intercepts for extraversion and negative emotionality were explained almost entirely by genetic influences. Accordingly, the observed between‐person phenotypic associations between extraversion and negative emotionality and peer affiliations were attributed to shared genetic influences. This suggests that inherited levels of extraversion and negative emotionality may help shape one's general attraction to, and attractiveness for, different friendship networks across adolescence. That is, friendly, outgoing youth may both prefer affiliating with prosocial peers, and face little trouble in joining such peer groups given both this nature and a comfort with social interaction. Alternatively, youth that are less pleasant, more aggressive, and less at‐ease in social interactions may have more trouble joining such prosocial peer groups, and may be less interested in doing so in any case.

In contrast, the link between personality traits associated with behavioral control and friends' antisocial and prosocial behaviors were attributable to both genetic and shared environmental influences. This indicates that in addition to the heritable mechanisms alluded to above, there are also shared environmental influences contributing to both a personality structure associated with greater behavioral control and adherence to norms and rules, and to forming friendships with peers that are more prosocial and less antisocial. These findings differ from expectations based on other behavior genetic studies, which have largely identified unique environmental effects as the critical environmental contributors to personality (Polderman et al., 2015). Identifying these shared environmental mechanisms could have a major impact on both positive personality development and social adjustment in adolescence. For example, home environments characterized by more emotional support and material resources may help push youth toward more prosocial peer groups and behavioral tendencies (either by parental involvement or sheer availability). Importantly, the results here are consistent with related studies about peer affiliations, personality, and sexual behaviors in adolescence, which have shown that shared environmental influences largely accounted for the association between friend behaviors and less normative sexual behaviors (e.g., early sexual debut; Clark et al., 2020), and between behavioral undercontrol and less normative sexual behaviors (Clark et al., 2021). Taken together, this implies a set of environmental presses that push youth toward behavioral undercontrol, antisocial peer affiliations, and risky sex behaviors. Identifying specific environmental influences here, and their relative importance across these domains is a critical avenue of future work.

### 4.3 Limitations and future directions

While this was a relatively unique analysis of the prospective associations between personality traits and friend characteristics across adolescence, the study had limitations. One, although our measure of peer characteristics captured norms and characteristics among friend groups, it did not assess the overall size, density, or quality of the relationships with friends, all important features of youths' social networks. Studies that identify the closeness or impact of different peers within the friendship network may help to better identify the processes that drive selection into these relationships and the processes by which these relationships influence personality. For example, a young person with a large social network that is on average quite prosocial could be particularly close with one antisocial best friend. Understanding how peer influences operate as a function of proximity, exposure, and intimacy are important questions to pursue.

Another potential limitation of the peer group measure was that the questionnaire relied on adolescents to rate their peers on antisocial and prosocial behaviors, and may thus function in part as proxy measures of youths' own behavior, leading to overestimates of how similar their peers' behaviors are to their own. A mitigating factor though was the use of teacher ratings of personality, which removed shared informant bias. Furthermore, while teachers who rated both members of a twin pair might inflate estimates of shared environmental influences on personality (though this situation was uncommon in this sample), this potential artifact cannot explain the shared environmental contribution to the link between personality traits (teacher report) and peer characteristics (twins' report) due to the different informant sources. Indeed, one strength using different informants for personality and peer group characteristics was an additional demonstration of the predictive power of teacher perceptions of youth traits for understanding individual differences in consequential socioemotional outcomes. The lack of racial and ethnic diversity in the sample was another major limitation of this study. Accordingly, caution is needed when attempting to generalize these findings as the trends identified here may differ in important ways when considering non‐White youth who come of age in a different sociocultural context that could exert different environmental pressures on personality and peer affiliations.

Future research can also identify the processes more proximally responsible for the impact of friendship characteristics on subsequent trait development, whether by seeking congruence with behavioral norms, behavioral change via identification with peers, punishment, and reinforcement of different patterns of behavior by friends, or the accumulated exposure to different micro‐environments as a result of time spent with antisocial or prosocial peers. To be sure, in our data, there remained substantial individual variability in the residual, within‐person structure after accounting for the cross‐lagged paths. This, along with the relatively modest autoregressive paths, suggests that not all children follow a pattern whereby traits and friendship groups converge in the same broad and internally coherent direction. Variability along these lines is yet another reason to consider more granular assessments of peer dynamics (e.g., whether these effects vary according to peer status or the fit versus misfit between adolescents' trait profile and the characteristics of their selected peer groups), and more tightly spaced assessment windows. Although it was possible to capture broad trends over time in the current data, the spacing between assessments precludes more focused conclusions regarding the processes whereby personality and friend groups affect one another. Studies that utilize longitudinal social network analysis to disentangle mechanisms of selection and influence will be especially useful for fleshing out the mechanisms driving associations between personality processes and friendship mechanisms (see Neal & Veenstra, 2021). Moreover, our analyses focused on how personality traits of youth are shaped by (and shape their selection into) peer groups, but do not consider how personality traits may shape which youth in a peer group have the power and ability to influence others; future research exploring these actor effects would be very important, particularly considering the role of status in shaping patterns of influence among adolescents.

## 5 CONCLUSION

We presented a thorough investigation spanning multiple levels of analysis that helped to reinforce and extend past work on the links between personality and peer groups in adolescence. The most robust findings lend further support to the notion that less adaptive trait profiles (i.e., high negative emotionality, low conscientiousness, and low agreeableness) are associated with more antisocial peer affiliations, largely due to both shared genetic *and* environmental influences. There was also some evidence for reciprocity and corresponsive processes such that traits in early adolescence help youth select into certain peer groups, which in turn reinforce these dispositional tendencies. Continuing to study the interplay between these person‐situation transactions, and genetic and environmental influences, is critical for understanding personality development across adolescence, and could ultimately produce information to help improve the adjustment of youth and prevent more serious negative outcomes in adulthood.

## AUTHOR CONTRIBUTIONS

D. Angus Clark, C. Emily Durbin, Mary M. Heitzeg, and Brian M. Hicks contributed to conception and design. William G. Iacono, Matt McGue, and Brian M. Hicks contributed to acquisition of data. D. Angus Clark, C. Emily Durbin, and Brian M. Hicks contributed to analysis and interpretation of data. D. Angus Clark, C. Emily Durbin, Mary M. Heitzeg, William G. Iacono, Matt McGue, and Brian M. Hicks drafted and/or revised the article. D. Angus Clark approved the submitted version for publication.

## ETHICS STATEMENT

All ethical guidelines for conducting research were followed in this study. Data collection was approved by the University of Minnesota institutional review board; parent consent and youth assent was obtained for all participants. The data file and sample syntax files for the methods used in the primary analyses can be found on the following OSF link: https://osf.io/9n7em/. The analyses here were not preregistered.

## FUNDING INFORMATION

This work was supported by the United States Public Health Service grants R01 AA09367 (McGue), R01 AA024433 (Hicks), and T32 AA007477 (Blow) from the National Institute of Alcohol Abuse and Alcoholism and R37 DA005147 (Iacono), R01 DA013240 (Iacono), and R01 DA039112 (Durbin) from the National Institute on Drug Abuse.

## CONFLICT OF INTEREST

The authors declare no competing interests.

## References

[jopy12741-bib-0002] Bleidorn, W. (2015). What accounts for personality maturation in early adulthood? Current Directions in Psychological Science, 24(3), 245–252. 10.1177/0963721414568662

[jopy12741-bib-0003] Borkenau, P. , Riemann, R. , Angleitner, A. , & Spinath, F. M. (2001). Genetic and environmental influences on observed personality: Evidence from the German Observational Study of Adult Twins. Journal of Personality and Social Psychology, 80(4), 655–668. 10.1037/0022-3514.80.4.655 11316228

[jopy12741-bib-0004] Briley, D. A. , Livengood, J. , & Derringer, J. (2018). Behaviour genetic frameworks of causal reasoning for personality psychology. European Journal of Personality, 32(3), 202–220. 10.1002/per.2153

[jopy12741-bib-0005] Briley, D. A. , Livengood, J. , Derringer, J. , Tucker‐Drob, E. M. , Fraley, R. C. , & Roberts, B. W. (2019). Interpreting behavior genetic models: Seven developmental processes to understand. Behavior Genetics, 49(2), 196–210. 10.1007/s10519-018-9939-6 30467668 PMC6904232

[jopy12741-bib-0065] Brown, B. B. (1999). "You're going out with who?": Peer group influences on adolescent romantic relationships. In W. Furman , B. B. Brown , & C. Feiring (Eds.), The development of romantic relationships in adolescence (pp. 291–329). Cambridge University Press. 10.1017/CBO9781316182185.013

[jopy12741-bib-0006] Brown, B. B. (2011). Popularity in peer group perspective: The role of status in adolescent peer systems. In A. H. N. Cillessen , D. Schwartz , & L. Mayeux (Eds.), Popularity in the peer system (pp. 165–192). Guilford Press.

[jopy12741-bib-0007] Brown, B. B. , & Klute, C. (2006). Friendships, cliques, and crowds. In G. R. Adams & M. D. Berzonsky (Eds.), Blackwell handbook of adolescence (pp. 330–348). Blackwell.

[jopy12741-bib-0008] Brown, B. B. , & Larson, J. (2009). Peer relationships in adolescence. In R. Lerner & L. Steinberg (Eds.), Handbook of adolescent psychology (pp. 74–103). Wiley & Sons.

[jopy12741-bib-0009] Burk, W. J. , Steglich, C. E. , & Snijders, T. A. (2007). Beyond dyadic interdependence: Actor‐oriented models for co‐evolving social networks and individual behaviors. International Journal of Behavioral Development, 31(4), 397–404. 10.1177/0165025407077762

[jopy12741-bib-0010] Burt, S. A. (2014). Research review: The shared environment as a key source of variability in child and adolescent psychopathology. Journal of Child Psychology and Psychiatry, 55(4), 304–312. 10.1111/jcpp.12173 24261560

[jopy12741-bib-0011] Cairns, R. B. , Cairns, B. D. , Neckerman, H. J. , Gest, S. D. , & Gariepy, J. L. (1988). Social networks and aggressive behavior: Peer support or peer rejection? Developmental Psychology, 24(6), 815–823. 10.1037/0012-1649.24.6.815

[jopy12741-bib-0013] Clark, D. A. , Durbin, C. E. , Heitzeg, M. M. , Iacono, W. G. , McGue, M. , & Hicks, B. M. (2020). Adolescent sexual development and peer groups: Reciprocal associations with shared genetic and environmental influences. Archives of Sexual Behavior, 50, PM32314108. 10.1007/s10508-020-01697-9 PMC8110336 32314108

[jopy12741-bib-0014] Clark, D. A. , Durbin, C. E. , Heitzeg, M. M. , Iacono, W. G. , McGue, M. , & Hicks, B. M. (2021). *Personality and sexual development in adolescence: Selection, corresponsive effects, and genetic and environmental influences*. Unpublished manuscript.

[jopy12741-bib-0015] Clark, D. A. , Durbin, C. E. , Hicks, B. M. , Iacono, W. G. , & McGue, M. (2017). Personality in the age of industry: Structure, heritability, and correlates of personality in middle childhood from the perspective of parents, teachers, and children. Journal of Research in Personality, 67, 132–143. 10.1016/j.jrp.2016.06.013 28408770 PMC5385923

[jopy12741-bib-0016] Coplan, R. J. , & Bullock, A. (2012). Temperament and peer relationships. In M. Zentner & R. L. Shiner (Eds.), Handbook of temperament (pp. 442–461). The Guilford Press.

[jopy12741-bib-0017] Cruz, J. E. , Emery, R. E. , & Turkheimer, E. (2012). Peer network drinking predicts increased alcohol use from adolescence to early adulthood after controlling for genetic and shared environmental selection. Developmental Psychology, 48(5), 1390–1402. 10.1037/a0027515 22390657 PMC3616641

[jopy12741-bib-0018] Denissen, J. J. , van Aken, M. A. , Penke, L. , & Wood, D. (2013). Self‐regulation underlies temperament and personality: An integrative developmental framework. Child Development Perspectives, 7(4), 255–260. 10.1111/cdep.12050

[jopy12741-bib-0020] Dishion, T. J. , & Owen, L. D. (2002). A longitudinal analysis of friendships and substance use: Bidirectional influence from adolescence to adulthood. Developmental Psychology, 38, 480–491. 10.1037/0012-1649.38.4.480 12090479

[jopy12741-bib-0019] Dishion, T. J. , Andrews, D. W. , & Crosby, L. (1995). Antisocial boys and their friends in early adolescence: Relationship characteristics, quality, and interactional process. Child Development, 66(1), 139–151. 10.1111/j.1467-8624.1995.tb00861.x 7497821

[jopy12741-bib-0021] Dishion, T. J. , Spracklen, K. M. , Andrews, D. W. , & Patterson, G. R. (1996). Deviancy training in male adolescent friendships. Behavior Therapy, 27(3), 373–390. 10.1016/S0005-7894(96)80023-2

[jopy12741-bib-0023] Durbin, C. E. , Hicks, B. M. , Blonigen, D. M. , Johnson, W. , Iacono, W. G. , & McGue, M. (2016). Personality trait change across late childhood to young adulthood: Evidence for nonlinearity and sex differences in change. European Journal of Personality, 30(1), 31–44. 10.1002/per.2013 26997753 PMC4795480

[jopy12741-bib-0024] Falk, C. F. (2018). Are robust standard errors the best approach for interval estimation with nonnormal data in structural equation modeling? Structural Equation Modeling: A Multidisciplinary Journal, 25(2), 244–266. 10.1080/10705511.2017.1367254

[jopy12741-bib-0025] Farrington, D. P. (1986). Age and crime. Crime and Justice, 7, 189–250. 10.1086/449114

[jopy12741-bib-0026] Farrington, D. P. (2004). Conduct disorder, aggression, and delinquency. In R. M. Lerner & L. Steinberg (Eds.), Handbook adolescent psychology (pp. 627–664). John Wiley & Sons Inc.

[jopy12741-bib-0027] Furman, W. , & Buhrmester, D. (1992). Age and sex differences in perceptions of networks of personal relationships. Child Development, 63(1), 103–115. 10.2307/1130905 1551320

[jopy12741-bib-0028] Gallupe, O. , Boman, J. H., IV , Nash, R. , & Castro, E. D. (2020). Deviant peer preferences: A simplified approach to account for peer selection effects. Deviant Behavior, 41(9), 1143–1156. 10.1080/01639625.2019.1597321 33299262 PMC7723346

[jopy12741-bib-0029] Hallquist, M. N. , & Wiley, J. F. (2018). MplusAutomation: An R package for facilitating large‐scale latent variable analyses in Mplus. Structural Equation Modeling: A Multidisciplinary Journal, 25(4), 621–638. 10.1080/10705511.2017.1402334 30083048 PMC6075832

[jopy12741-bib-0030] Hamaker, E. L. , Kuiper, R. M. , & Grasman, R. P. (2015). A critique of the cross‐lagged panel model. Psychological Methods, 20(1), 102–116. 10.1037/a0038889 25822208

[jopy12741-bib-0031] Hartup, W. W. , & Stevens, N. (1997). Friendships and adaptation in the life course. Psychological Bulletin, 121(3), 355–370. 10.1037/0033-2909.121.3.355

[jopy12741-bib-0032] Iacono, W. G. , Carlson, S. R. , Taylor, J. , Elkins, I. J. , & McGue, M. (1999). Behavioral disinhibition and the development of substance use disorders: Findings from the Minnesota twin family study. Development and Psychopathology, 11, 869–900. 10.1017/S0954579499002369 10624730

[jopy12741-bib-0068] Johnson, A. M. , Vernon, P. A. , & Feiler, A. R. (2008). Behavioral genetic studies of personality: An introduction and review of the results of 50+ years of research. The SAGE handbook of personality theory and assessment, 1, 145–173.

[jopy12741-bib-0034] Kandler, C. , & Zapko‐Willmes, A. (2017). Theoretical perspectives on the interplay of nature and nurture in personality development. In Personality development across the lifespan (pp. 101–115). Academic Press.

[jopy12741-bib-0033] Kandler, C. , Waaktaar, T. , Mõttus, R. , Riemann, R. , & Torgersen, S. (2019). Unravelling the interplay between genetic and environmental contributions in the unfolding of personality differences from early adolescence to young adulthood. European Journal of Personality, 33(3), 221–244. 10.1002/per.2189

[jopy12741-bib-0035] Keyes, M. A. , Malone, S. M. , Elkins, I. J. , Legrand, L. N. , McGue, M. , & Iacono, W. G. (2009). The Enrichment Study of the Minnesota Twin Family Study: Increasing the yield of twin families at high risk for externalizing psychopathology. Twin Research and Human Genetics, 12(5), 489–501. 10.1375/twin.12.5.489 19803776 PMC2760025

[jopy12741-bib-0036] Laninga‐Wijnen, L. , & Veenstra, R. (2021). Peer similarity in adolescent social networks: Types of selection, influence, and factors contribution to openness to peer influence. In B. Halpern‐Felsher (Ed.), The encyclopedia of child and adolescent health (pp. 1‐39). Elsevier.

[jopy12741-bib-0037] Laursen, B. , & Veenstra, R. (2021). Toward understanding the functions of peer influence: a summary and synthesis of recent empirical research. Journal of Research on Adolescence, 31(4), 889–907. 10.1111/jora.12606 34820944 PMC8630732

[jopy12741-bib-0038] Li, Y. , Doyle Lynch, A. , Kalvin, C. , Liu, J. , & Lerner, R. M. (2011). Peer relationships as a context for the development of school engagement during early adolescence. International Journal of Behavioral Development, 35(4), 329–342.

[jopy12741-bib-0039] Lodder, G. M. , Scholte, R. H. , Cillessen, A. H. , & Giletta, M. (2016). Bully victimization: Selection and influence within adolescent friendship networks and cliques. Journal of Youth and Adolescence, 45(1), 132–144. 10.1007/s10964-015-0343-8 26323168 PMC4698289

[jopy12741-bib-0069] McGue, M. , Zhang, Y. , Miller, M. B. , Basu, S. , Vrieze, S. , Hicks, B. , Malone, S. , Oetting, W. S. , & Iacono, W. G. (2013). A genome‐wide association study of behavioral disinhibition. Behavior Genetics, 43(5), 363–373. 10.1007/s10519-013-9606-x 23942779 PMC3886341

[jopy12741-bib-0040] McPherson, M. , Smith‐Lovin, L. , & Cook, J. M. (2001). Birds of a feather: Homophily in social networks. Annual Review of Sociology, 27(1), 415–444. 10.1146/annurev.soc.27.1.415

[jopy12741-bib-0042] Monahan, K. C. , Steinberg, L. , & Cauffman, E. (2009). Affiliation with antisocial peers, susceptibility to peer influence, and antisocial behavior during the transition to adulthood. Developmental Psychology, 45(6), 1520–1530. 10.1037/a0017417 19899911 PMC2886974

[jopy12741-bib-0044] Muthen, L. K. , & Muthen, B. O. (1998‐2018). Mplus user's guide (8th ed.). Muthen & Muthen.

[jopy12741-bib-0043] Mõttus, R. , Briley, D. A. , Zheng, A. , Mann, F. D. , Engelhardt, L. E. , Tackett, J. L. , Harden, K. P. , & Tucker‐Drob, E. M. (2019). Kids becoming less alike: A behavioral genetic analysis of developmental increases in personality variance from childhood to adolescence. Journal of Personality and Social Psychology, 117(3), 635–658. 10.1037/pspp0000194 30920282 PMC6687565

[jopy12741-bib-0046] Neal, J. W. , & Veenstra, R. (2021). Network selection and influence effects on children's and adolescents' internalizing behaviors and peer victimization: A systematic review. Developmental Review, 59, 100944.

[jopy12741-bib-0045] Neal, J. W. , Neal, Z. P. , & Cappella, E. (2014). I know who my friends are, but do you? Predictors of self‐reported and peer‐inferred relationships. Child Development, 85(4), 1366–1372. 10.1111/cdev.12194 24320155 PMC4050041

[jopy12741-bib-0047] Polderman, T. J. , Benyamin, B. , De Leeuw, C. A. , Sullivan, P. F. , Van Bochoven, A. , Visscher, P. M. , & Posthuma, D. (2015). Meta‐analysis of the heritability of human traits based on fifty years of twin studies. Nature Genetics, 47(7), 702–709. 10.1038/ng.3285 25985137

[jopy12741-bib-0048] Prescott, C. A. (2004). Using the Mplus computer program to estimate models for continuous and categorical data from twins. Behavior Genetics, 34(1), 17–70. 10.1023/B:BEGE.0000009474.97649.2f 14739694

[jopy12741-bib-0049] Prinstein, M. J. , & Giletta, M. (2016). Peer relations and developmental psychopathology. In D. Cicchetti (Ed.), Developmental psychopathology: Vol. 1. Theory and method (pp. 527–579). Wiley.

[jopy12741-bib-0050] R Core Team . (2016). R: A language and environment for statistical computing (3.3.1) [computer program]. R Foundation for Statistical Computing.

[jopy12741-bib-0052] Roberts, B. W. , Caspi, A. , & Moffit, T. E. (2003). Work experiences and personality development in young development. Journal of Personality and Social Psychology, 74, 582–593. 12635918

[jopy12741-bib-0054] Robin, S. S. , & Johnson, E. O. (1996). Attitude and peer cross pressure: Adolescent drug and alcohol use. Journal of Drug Education, 26, 69–99. 10.2190/W8QF-V3KF-N9C3-86UE 8991971

[jopy12741-bib-0055] Rubin, K. H. , Bowker, J. C. , McDonald, K. L. , & Menzer, M. (2013). Peer relationships in childhood. In P. D. Zelazo (Ed.), The Oxford handbook of developmental psychology, Vol. 2: Self and other (Vol. 2, pp. 242–275). Oxford University Press.

[jopy12741-bib-0056] Scarr, S. , & McCartney, K. (1983). How people make their own environments: A theory of genotype→ environment effects. Child Development, 54, 424–435. 6683622 10.1111/j.1467-8624.1983.tb03884.x

[jopy12741-bib-0066] Soto, C. J. (2015). Is happiness good for your personality? Concurrent and prospective relations of the big five with subjective well‐being. Journal of personality, 83(1), 45–55. 24299053 10.1111/jopy.12081

[jopy12741-bib-0058] Soto, C. J. , John, O. P. , Gosling, S. D. , & Potter, J. (2011). Age differences in personality traits from 10 to 65: Big Five domains and facets in a large cross‐sectional sample. Journal of Personality and Social Psychology, 100(2), 330–348. 10.1037/a0021717 21171787

[jopy12741-bib-0059] Tellegen, A. , & Waller, N. G. (2008). Exploring personality through test construction: Development of the Multidimensional Personality Questionnaire. In G. J. Boyle , G. Matthews , & D. H. Saklofske (Eds.), The SAGE handbook of personality theory and assessment (Vol. 2, pp. 261–292). Sage Publications, Inc.

[jopy12741-bib-0061] Walden, B. , McGue, M. , Burt, S. A. , & Elkins, I. (2004). Identifying shared environmental contributions to early substance use: the respective roles of peers and parents. Journal of Abnormal Psychology, 113(3), 440–450. 10.1037/0021-843X.113.3.440 15311989

[jopy12741-bib-0062] West, S. G. , Taylor, A. B. , & Wu, W. (2012). Model fit and model selection in structural equation modeling. In R. H. Hoyle (Ed.), Handbook of structural equation modeling (Vol. 1, pp. 209–231). Guilford Press.

[jopy12741-bib-0063] Wrzus, C. , & Neyer, F. J. (2016). Co‐development of personality and friendships across the lifespan: An empirical review on selection and socialization. European Psychologist, 21(4), 254–273. 10.1027/1016-9040/a000277

[jopy12741-bib-0067] Zimmermann, J. & Reitz, A. , (2018). In: V. Zeigler‐Hill & T. Shackelford (Eds.), The SAGE handbook of personality and individual differences (1st ed). Sage Publishers.

